# The Expanding Scope of Alpha 1 Antitrypsin Deficiency

**DOI:** 10.1016/j.mayocpiqo.2023.12.001

**Published:** 2024-01-06

**Authors:** Friedrich Kueppers

**Affiliations:** Department of Thoracic Medicine and Surgery, Lewis Katz School of Medicine at Temple University, Philadelphia, PA

Alpha 1 antitrypsin deficiency (AATD) is a hereditary disorder first described in 1963 as a genetic cause of chronic obstructive pulmonary disease (COPD).^1^ AATD is characterized by low levels of alpha 1 antitrypsin (AAT) allowing neutrophil elastase (NE) to destroy the elastin structure of the lung.^2^ Although the major function of AAT as a protease or elastase inhibitor (PI) has attracted the most attention, anti-inflammatory, and immune-regulatory activity, possibly unrelated to its PI activity, have also been suggested.^3^^,^^4^

AAT is encoded by the *SERPINA1* gene on chromosome 14. The *SERPINA1* is highly polymorphic and different mutations have a range of pathological implications.^5^^,^^6^ Individuals homozygous for the normal M allele (PI∗MM) typically have serum AAT levels of 17-47 μM (102-254 mg/dL), whereas individuals homozygous for the Z allele (PI∗ZZ), which is associated with severe AATD, have serum AAT levels <10 μM (<52 mg/dL).^7^ Although other variants and genotypes are associated with varying serum AAT levels, other subvariants are common, such as M1, M2, M3, and M4.^5^ These subvariants were revealed by further analysis of the original M classification by Fagerhol in 1969.^8^ Recent literature suggests that the M3 variant can contribute to the pathogenesis of COPD and other disorders by mechanisms that warrant further investigation.^2^ Therefore, in addition to increased susceptibility to lung degradation, AATD also contributes to the susceptibility to other disorders.

As an acute phase reactive protein, AAT has a range of anti-inflammatory and immunomodulatory properties.^2^ Due to the systemic nature of AAT, many extrapulmonary manifestations have previously been linked to AATD; several of which are associated with the Z variant, such as liver disease, panniculitis, and vasculitis.^2^ In addition to these established extrapulmonary manifestations, recent evidence described below suggests that lower than normal AAT levels could be a genetic risk factor for vascular changes that could result in cardiovascular pathologies, such as aortic aneurysms.

### Vascular Changes

Vascular changes can be explained by uncontrolled proteolytic or elastalytic action on the vascular wall.^2^ The pathology is likely to be explained by the direct effect of increased NE activity due to reduced levels of its principal regulator, AAT. Ebert et al^9^ describes a novel mechanism of AAT binding to proteinase 3 (PR3), which in turn prevents PR3 binding and activation of neutrophils by the neutrophil surface receptor (CD177). In addition, the overactivation of NE is associated with amplified metalloproteases (MMPs), specifically MMP-9.^10^ MMPs are synthesized and excreted as precursor inactive zymogens that are activated by proteolytic enzymes, such as NE, and act as catalysts for the breakdown of the extracellular matrix (ECM).^11^ Overactivity of NE due to reduced AAT levels, as seen in individuals with AATD, leads to the amplification of MMP-9 and an increase in destructive action on elastin and the ECM in the aortic wall. Increased levels of MMP-9 have been identified in the vessel wall of aortic aneurysms and are correlated with aneurysm diameter,^12^ supporting the argument that MMP-9 has a key role in the development of aneurysms resulting from decreased serum AAT levels. This relationship is summarized in Figure 1.

**Figure 1:**
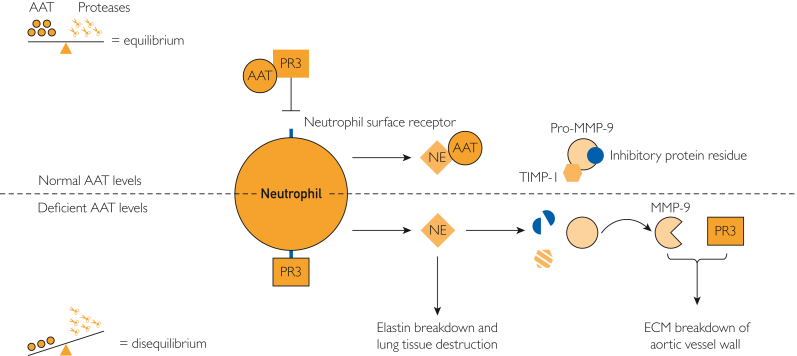
Mechanism of action reporting that decreased serum AAT can result increased destructive action on elastin and the aortic ECM. AAT, alpha 1 antitrypsin; AATD, alpha 1 antitrypsin deficiency; ECM, extracellular matrix; MMP-9, metalloprotease-9; NE, neutrophil elastase; PR3, proteinase 3; TIMP-1, tissue inhibitor of matrix metalloproteinases-1.

As shown in Figure 1, with normal levels of AAT, there is a balance in protease/anti-protease activity. AAT inhibits NE activity on elastin within the lung tissue; the binding of AAT to PR3 prevents PR3 binding to the neutrophil surface receptor,^9^ which in turn prevents the release of NE by activated neutrophils and reduces levels of active MMP-9.^10^^,^^11^ In AATD, deficient levels of AAT result in a protease/anti-protease imbalance. Excess PR3 can bind to the neutrophil surface receptor, leading to the release of NE by the activated neutrophil. The NE primarily degrades elastin but can also cleave the inhibitory protein residue on MMP-9 and degrade the MMP-9 inhibitor (tissue inhibitor of matrix metalloproteinases-1), which leads to persistent MMP activity and aortic vessel wall ECM degradation.^10^

### Aneurysmal Disease

In patients with AATD, the lack of NE inhibition and overactivity of proteases can degrade elastin and connective tissues, leading to a loss of elasticity in vessel walls, increased stiffness, and reduced distensibility. This can result in aortic wall weakening and aortic distention.^5^^,^^13^ There is an abundance of evidence reporting the protective role of AAT in preserving arterial wall integrity and regulation of inflammatory processes underlying cardiac events. For example, Pini et al^14^ describes multiple studies, which report that defective forms of AAT contribute to a range of cardiovascular diseases, resulting from the imbalance of protease and anti-protease that is associated with AATD. Although data regarding the potential association of AATD and ascending aortic aneurysm are limited, a pathological association between AATD and aortic wall distension with a subsequent increased risk for aneurysm development has been previously documented.^13^

The first controlled study to assess the relationship between AATD and ascending aortic diameter has recently been reported. This single-center study retrospectively collected data on patients with AATD-associated emphysema and non-AATD-associated emphysema.^13^ Data reported there was a significant positive correlation between ascending aortic diameter and age in patients with AATD, which was not observed in the non-AATD-associated emphysema control group (Figure 2).^13^ Serum AAT levels of the study group were ∼9.5 times lower than those in the control group, indicating that reduced serum AAT levels are pathologically associated with arterial distention in patients with AATD and that arterial distention risk increases with increasing age. For patients with AATD, a significant positive correlation was observed between aortic diameter and age (*r*=0.43; *P*=.0016).^13^ This relationship was not observed in the control group (*r*=0.16; *P*=.11).^13^

**Figure 2:**
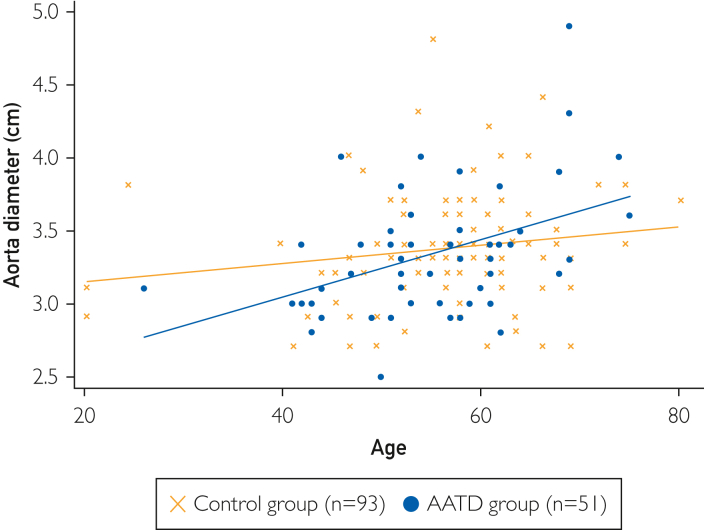
Correlations between age and aortic diameter in the AATD and control groups in the AATD aortic distention study. Reproduced with permission from Dako et al.^13^ AATD, alpha 1 antitrypsin deficiency.

The vascular pathology associated with AATD can take several forms that occur in different vessels, and in the same artery at different levels. Evidence suggests that specific AATD genotypes could be associated with the aneurysm location. For example, heterozygous genotypes, specifically with the S allele, have been associated with abdominal aortic aneurysms,^15^ whereas the homozygous PI∗ZZ genotype has been associated with ascending aortic aneurysm.^13^ In addition, observational studies have shown a higher incidence of cerebral aneurysms in patients with heterozygous and homozygous AATD genotypes compared with the general population.^16^^,^^17^

We can also report the relevance of vascular pathology and AATD with current clinical history of patients with emphysema and AATD, specifically with the additional presence of aneurysmal disease. Current ongoing research highlighted patients who, in addition to AATD, had extensive panacinar emphysema and a history of vascular pathology, and were identified for inclusion in a case report. A 70-year-old White female (patient 1) who suffered a ruptured aneurysm of the splenic artery in 2018, and a 52-year-old White male who suffered a dissection of the descending aorta in 2017 (patient 2). For patient 1, the aneurysm was successfully coiled and for patient 2, the dissection was successfully treated with an endovascular stent. Both patients are doing well and in addition to their conventional COPD treatment, are currently receiving AAT therapy. Their detailed clinical history and documentation will be published separately (manuscript in preparation).

### New and Emerging Treatments

Alternatives to intravenous AAT therapy are currently being investigated with different modes of action. Newer cost-effective treatments may encourage further research for AATD that focus on targeting areas beyond disease progression and reducing symptom severity. Preclinical and clinical studies have investigated several approaches and gene therapy appears to be the most promising.^18^ Advances in gene therapy for AATD include gene repair (hepatocyte replacement, clustered regularly interspaced short palindromic repeat [CRISPR] strategies, and recombinant adeno-associated virus vectors), RNA interference strategies, and the use of biomarkers, and chemical chaperones using *in silico* ligand screening.^19^ The first *in vivo* gene therapy for treating patients with AATD was conducted in animals over 30 years ago; however, there is still no Food and Drug Administration (FDA)-approved gene therapy for AATD.^20^^,^^21^

CRISPR technology has been revolutionizing research for the treatment of many genetic diseases. In AATD, current treatment strategies are employing this technology to simultaneously silence the Z allele and express the M allele of *SERPINA1*.^18^^,^^19^ This strategy would prevent expression of misfolded AAT proteins responsible for inducing liver disease, and allow normal AAT proteins to be secreted into the circulatory system at normal levels and alleviate lung disease. CRISPR technology therefore represents a new and exciting approach that could solve both lung and liver manifestations of AATD, and may also correct the associated aneurysmal disease.^19^

## Conclusion

There is an abundance of evidence reporting the protective role of AAT in preserving arterial wall integrity and the regulation of inflammatory processes. Reduced AAT levels result in overactive proteases, leading to the amplification of MMPs such as MMP-9, and the increased destructive action on the ECM and elastin of the aortic wall.

Data show there is a pathological association between AAT and the development of aortic distention, with age as a driving factor. AAT is genetically highly polymorphic with multiple alleles influencing serum levels, and its functionality. A pathological association is evident between different AAT alleles, with respect to vascular disease and the coexistence of emphysema, as well as aortic and cerebral aneurysms. It is, therefore, important to consider the expanding scope of AATD, and patients with this condition should be monitored for cardiovascular implications.

## Potential Competing Interests

The author declares no conflict of interest in preparing this article.
